# Understanding the Association between PrEP Stigma and PrEP Cascade Moderated by the Intensity of HIV Testing

**DOI:** 10.3390/tropicalmed7050074

**Published:** 2022-05-16

**Authors:** Chen Zhang, Yu Liu

**Affiliations:** 1 School of Nursing, University of Rochester Medical Center, Rochester, NY 14642, USA; 2 Department of Public Health Science, School of Medicine and Dentistry, Rochester, NY 14642, USA; yu_liu@urmc.rochester.edu

**Keywords:** men who have sex with men, pre-exposure prophylaxis, PrEP stigma, United States, HIV testing

## Abstract

(1) Background: In the U.S., men who have sex with men (MSM) account for the majority of new HIV infections. On the other hand, pre-exposure prophylaxis (PrEP) is an effective strategy to curb HIV transmission, but it is widely underutilized. It is unknown how stigma affects PrEP care in the context of other HIV prevention strategies. (2) Methods: We included a total of 318 MSM in the current analysis. We employed bivariate and multivariable analyses to assess the association between PrEP stigma and PrEP cascade while controlling for potential confounders on each specific pathway. We further used a series of moderation analyses based upon the intensity of HIV testing within different timeframes to assess the association between PrEP stigma and PrEP cascade. (3) Results: Compared with MSM who used PrEP, those who never used PrEP reported higher internalized and vicarious PrEP stigma. Internalized PrEP stigma has significantly reduced the likelihood of PrEP willingness and PrEP uptake among this group. The trend analysis showed significant trend patterns across different frequencies of HIV testing. (4) Conclusions: A structural-level reform is urgently needed to turn the HIV service encounters into opportunities to facilitate and optimize the PrEP cascade among this group who may benefit from PrEP use.

## 1. Introduction

Men who have sex with men (MSM) are the most disproportionally burdened sexual minority by HIV [1,2]. Although targeted efforts have stabilized new HIV diagnoses and increased awareness of HIV status, MSM continue to be the most affected, accounting for 69% of newly HIV-diagnosed infections in 2018 in the United States [1,2]. The burden is exceptionally high for young MSM who disproportionately experience HIV diagnosis, suboptimal linkage to care, and HIV prevention services [3,4,5].

HIV Prevention Continuum Framework conceptualizes the specific steps (i.e., HIV testing, linkage to prevention services, retention in services, and adherence support) along the HIV prevention spectrum [6]. As the critical components of the Framework, HIV testing and pre-exposure prophylaxis (PrEP) are interconnected and play critical roles in the prevention spectrum [6]. As a gateway to initiating the continuum, HIV testing determines the direction of the following path. It is recommended that MSM at elevated HIV risks undergo HIV testing every 3–6 months [1]. With a negative HIV testing result, an uninfected person with HIV risk will be linked with and retained in HIV prevention services, such as PrEP care. With the ongoing engagement in the prevention process, the person will be retained in the prevention continuum as long as the risk remains to maintain the desired protective effect [6]. Furthermore, PrEP is an efficacious HIV prevention strategy when taken as prescribed, especially among MSM [7].

Despite HIV testing and PrEP use having been considered crucial tools for ending the epidemic, HIV testing rates among MSM remain suboptimal in the United States [8]. Besides, a significant discrepancy was observed from lifetime HIV testing (i.e., 88.2%) to frequent HIV testing (i.e., 42.2%) [9]. Furthermore, little data were available to assess the PrEP linkage and uptake rates initiated from HIV testing programs [10]. Although an upward trend in PrEP use has been observed among MSM across the years, inadequate PrEP knowledge, limited awareness, and low uptake have been observed continuously [11].

Unique barriers to the HIV prevention continuum have been identified among MSM, such as poverty, mental health status, access to healthcare, and insurance coverage [9,11]. HIV or PrEP-related stigma also plays a crucial role in preventing MSM from seeking HIV care [2,7,12,13]. Even for individuals who are actively seeking HIV prevention services (e.g., HIV testing, linkage to PrEP), the internalized (e.g., self-perceived stigmatization) and vicarious stigma (e.g., stigmatized attitudes from health providers or significant others) impede them from accessing HIV prevention care, such as PrEP [14].

Studies suggested that MSM receiving more intensive HIV testing services were more likely to engage in safer sex practices (e.g., condom use) than those who received less intensive services [15,16]. Moreover, the impact of HIV testing services on risk reduction behaviors (e.g., frequency of condom use, multi-partnership) is more substantial among HIV-positive MSM than among HIV-negative ones [17]. HIV testing services may represent an opportunity to enhance the HIV prevention care continuum. However, it is understudied how HIV testing services moderate the association between PrEP/HIV stigma and PrEP care linkage and uptake [17]. In the current study, we explored how PrEP and HIV-related stigma affect the PrEP care cascade (i.e., awareness, willingness, and uptake) and how the intensity of HIV testing services moderates the association. We hypothesized that the more a man received HIV testing services, the more likely he engaged in PrEP care.

## 2. Materials and Methods

Study Design and Participants: The sampling and recruiting strategies have been documented in detail elsewhere [18,19]. Briefly, between 2019 May and 2020 May, we conducted a cross-sectional study in two cities (Nashville, Tennessee, and Buffalo, New York) in the United States. We employed a multi-pronged recruiting strategy including peer referral, flyer distribution, social media posts, and venue-based and event-based recruitment. Eligible participants were those who (a) aged between 18 to 35 years old; (b) resided in either Nashville or Buffalo; (c) self-identified as gay, bisexual, and a man who had sex with another man in the past year; (d) self-reported HIV status as either negative or unknown; (e) were able to communicate in English and (f) provided informed consent. The study protocol was reviewed and approved by the Institutional Review Boards at the University of Rochester and Buffalo.

Data collection and Measures: Participants were asked to complete a self-administered survey via the Research Electronic Data Capture (REDCap) to report data regarding demographics, sexual behaviors, history of substance use, HIV testing experience, mental health status, PrEP/HIV related stigma, and PrEP cascade (i.e., awareness, willingness, and uptake). There were two options available for participants: they can either complete the survey at the research site using a provided laptop or tablet, or they can request the research coordinators to send them a secure survey link with an access code to the provided email address to complete the survey at a different time or location. Each participant who completed the survey received a gift card of $35 as a token of appreciation.

Demographics included age (in years), race (Black vs. White), education level, housing status (stable vs. unstable), insurance coverage, and marital status. Risk behaviors were measured by sexual practice (e.g., condomless insertive or receptive sex, sex with HIV-positive partners, substance use during sex, and sex position). A series of indicators measured mental health status (see Appendix A). Anxiety was assessed using the seven-item Generalized Anxiety Disorder Assessment Scale (e.g., “Have you been feeling nervous, anxious, or on edge in the past four weeks?”; Cronbach’s α = 0.93) [20]. Depression was measured using the 9-item Patient Health Questionnaire (e.g., “In the past four weeks, how often did you feel little interest or pleasure in doing things?”; Cronbach’s α = 0.94) [21,22]. Loneliness was measured using the University of California at Los Angeles Loneliness Scale (e.g., “I feel left out”; Cronbach’s α = 0.80) [23]. Perceived stress was assessed using the 10-item Perceived Stress Scale that measures stress in the past four weeks (e.g., “how often have you been upset because of something that happened unexpectedly?”; Cronbach’s α = 0.89) [24]. Suicide was measured using a four-question scale adapted from validated studies (e.g., “have you ever thought about or attempted to kill yourself?”; Cronbach’s α = 0.83) [18,25]. Internalized homophobia was measured by a four-item Internalized Homophobia Scale that measures the extent to which gay and bisexual individuals do not accept their sexual orientation or sexual identity (e.g., “Sometimes I dislike myself for being gay or bisexual”; Cronbach’s α = 0.91) [26]. Resilience was measured by the 10-item Conner–Davidson Resilience Scale (e.g., “I am able to adapt to change”, Cronbach’s α = 0.88) [27,28]. The Condom use Self-Efficacy Scale measured confidence in condom use to assess one’s confidence with using a condom or asking sexual partners to use condoms (e.g., “I would feel comfortable discussing condom use with a potential partner before we engaged in sex.”; Cronbach’s α = 0.88) [29,30]. HIV testing self-efficacy (e.g., “Knowing where you can go for an HIV test”, “Getting tested for HIV at least every 3–6 months”; Cronbach’s α = 0.91) to ask them how confident they were they about enacting behaviors concerning HIV testing [31]. HIV testing was measured by asking participants whether they had (yes vs. no) tested for HIV in the past 3, 6, or 12 months. PrEP and HIV related stigma was measured by internalized PrEP stigma (e.g., “I should avoid taking PrEP because it is only for slutty people”; Cronbach’s α = 0.93), vicarious PrEP stigma (e.g., “I’ve seen/heard people not wanting to hang out with folks who are taking PrEP”; Cronbach’s α = 0.93) and perceived HIV stigma toward MSM (e.g., “People I care about would stop being in touch with me after if I had HIV”; Cronbach’s α = 0.94) that was adopted from a recent study [32]. PrEP cascade was assessed using PrEP awareness, willingness to PrEP use in general and in specific scenarios (e.g., “If PrEP may cause mild side effects, such as nausea, headaches, and rashes in a small number of people, would still you take PrEP every day so you can lower your HIV risk by 90%?”, “If you need to see a clinician every 3–6 months for a new prescription, would you still consider taking PrEP every day to lower your HIV risk by 90%?”), and PrEP uptake (i.e., ever used and currently using). This study used indicators along the PrEP cascade (i.e., PrEP awareness, willingness, and uptake) as dependent variables.

Sample Size Calculation and Statistical Analysis: Assuming a past-6-month HIV testing rate of 70% among YBMSM [33], the minimum sample size to detect the prevalence is (1.96^2^ × 0.7 × (1-0.7))/0.05^2^ = 323 [34]. In the current study, we included 318 MSM in the analysis, which provided a marginally sufficient sample size to detect trends in the data. Descriptive statistics were displayed for both continuous and categorical variables. We used Chi-square and Independent *t*-tests to examine if demographics, risk behaviors, and mental health status varied at different stages of the PrEP cascade. We employed bivariate and multivariable analyses to assess the association between PrEP stigma and PrEP cascade while controlling for potential confounders on each specific pathway. Furthermore, a series of moderation analyses were conducted using Hayes’ PROCESS macro [35] with 2000 times bootstrapping samples to assess the effect of frequency of HIV testing under different timeframes (e.g., in the past 3, 6, 12, and 24 months) on the association between PrEP/HIV stigma and PrEP cascade. Adjusted odds ratios and corresponding 95% confidence intervals were reported. In addition, trend analyses using the Jonckheere–Terpstra test with Monte Carlo permutations were used to assess the trend of PrEP cascade across different time points of HIV testing. Furthermore, we provided the Cronbach’s α for each included scale, which measured the reliability or internal consistency of the scale. We calculated Cronbach’s α by comparing the average covariance between any pair of items with the variance of the total score of the assessed scale [36]. We conducted all statistical analyses using Stata 16.0TM (StataCorp LP, College Station, TX, USA).

## 3. Results

### 3.1. Participants’ Characteristics

A total of 318 MSM who self-reported as Black (*n* = 209) or White (*n* = 109) were included in the current analysis. The mean age of included participants was 25.86 years old (standard deviation [SD] = 4.94), and 74.21% of them lived in Nashville, Tennessee, and 25.79% lived in Buffalo, New York. The majority of them had some college degree or above (80.51%), reported insurance coverage (81.76%), had stable housing (75.79%) and were never married (90.57%). Among these MSM, 60.32% reported having condomless insertive sex, and 56.27% reported having condomless receptive sex. About one-quarter of them reported having sex with HIV-positive partners, 58.49% reported alcohol use during sex, and 39.49% reported recreational drug use during sex. MSM who were older, White, and had stable housing reported a higher prevalence of PrEP awareness and PrEP uptake than their peers. In addition, MSM who reported risk behaviors were more likely to report PrEP awareness, willingness, and uptake than those who did not. Furthermore, MSM who reported worse depression and loneliness conditions, had better resilience, confidence in condom use, and HIV testing self-efficacy were more likely to report PrEP awareness and willingness and uptake (Table 1 and Table 2).

### 3.2. Associations between PrEP Stigma and PrEP Cascade

Compared with MSM who used PrEP, those who never used PrEP reported higher internalized (0.59 vs. 2.99) and vicarious PrEP stigma (3.66 vs. 5.56). MSM who reported higher perceived HIV stigma towards MSM usually had a higher prevalence of PrEP awareness (29.65 vs. 26.33). While controlling for confounders on these pathways, internalized stigma significantly reduced the odds of being willing to use PrEP under various hypothetical scenarios, such as if PrEP may cause mild side effects (adjusted odds ratios [aOR] = 0.90, 95% confidence interval [CI] = 0.83, 0.97), if need to see a clinician every 3–6 months for a new prescription (aOR = 0.90, 95% CI = 0.83, 0.97), if need to get a blood test every 3–6 months to check if the pill has affected your kidney function (aOR = 0.89, 95% CI = 0.82, 0.97), if need to get a regular HIV test every 3–6 months to determine your eligibility for PrEP (aOR = 0.88, 95% CI = 0.81, 0.96), if it will not work well if do not use it daily (aOR = 0.92, 95% CI = 0.85, 0.99), and if a friend or your partner(s) finds out you are taking PrEP and might suggest you are at risk for HIV (aOR = 0.84, 95% CI = 0.76, 0.92). The internalized stigma also reduced the odds of ever using PrEP by 24% (aOR = 0.76, 95% CI = 0.65, 0.89). Besides, mixed findings were identified for vicarious stigma and PrEP HIV stigma towards MSM on the PrEP cascade (Table 3).

### 3.3. Moderation Effects by Frequency of HIV Testing under Different Timeframes

We conducted a series of moderation analyses based on HIV testing at different timeframes (i.e., in the past 3-month, 6-month, 12-month, and 24-month). With the increased frequency of HIV testing, the effect of PrEP stigma became stronger to impact the likelihood of PrEP willingness and PrEP uptake negatively. Specifically, in the past 24-month HIV testing history, for every one-unit increase in PrEP stigma, the odds of reporting willingness to PrEP use gradually decreased from 0.97 (95% CI = 0.93, 0.99) when testing four times, to 0.94 (95% CI = 0.89, 0.99) when testing six times, to 0.91 (95% CI = 0.84, 0.98) when testing eight times. Similarly, the odds gradually decreased from 0.77 (95% CI = 0.65, 0.91) when testing two times, 0.70 (95% CI = 0.56, 0.87) when testing four times, 0.63 (95% CI = 0.44, 0.90) when testing six times, and to 0.57 (95% CI = 0.34, 0.95) when testing eight times. The Jonckheere–Terpstra test with Monte Carlo permutations showed significant trend patterns across different frequencies of HIV testing. The same patterns have been identified under other timeframes (i.e., in the past 12-month, 6-month, and 3-month) (Table 4).

## 4. Discussion

To our knowledge, the current study is the first one to assess the moderation effect of intensity of HIV testing on the association between PrEP stigma and PrEP cascade among MSM in the United States. Consistent with the literature, PrEP stigma remains a formidable barrier to engaging in the PrEP cascade by discouraging people from seeking information, care, and support, preventing them from getting tested, and linking and retaining in care [37]. For instance, in a Demo project in San Francisco, MSM reported a feeling of being stigmatized by their significant others (e.g., sex partners, friends, and health providers) due to their decision to start using PrEP [38]. These stigmatized feelings and experiences hindered MSM from accessing and engaging in care, leading to a reduced quality of care and deteriorated intervention effectiveness [39,40].

Contrary to our hypothesis, findings indicated that the increased intensity of HIV testing services might discourage MSM from PrEP uptake or willingness to use it. With the higher intensity of HIV testing, MSM who encounter stigma were less likely to use or be willing to use PrEP. The same patterns have been observed under different periods (i.e., in the past 24-, 12-, 9-, 6-, and 3-month timeframe). Several reasons may explain the observed but unexpected phenomenon. First, studies revealed that MSM might have HIV testing experience in an unfriendly testing environment created by health professionals [37,41]. The hostile feeling was strong in young and racial/ethnic minority MSM [41,42,43]. The hostile environment discouraged MSM from engaging in other HIV prevention services, including PrEP care [37,41]. Second, some MSM may use frequent HIV testing as a strategy for HIV prevention. Instead of using PrEP, MSM use HIV testing to assess their risks. Furthermore, the cost of HIV testing is usually much lower than the cost of taking PrEP. Third, scarcity of comprehensive support for MSM was identified as a barrier for MSM engaging with PrEP care. Some MSM reported a lack of PrEP education and PrEP care navigation in HIV testing centers. MSM may not be aware of PrEP availability or clear about the PrEP care procedure [41]. Lastly, there may be a lack of provider knowledge preventing MSM from getting recommendations for PrEP care in HIV testing sessions [44]. In contrast, providers may play a key role in comparing the pros and cons of PrEP uptake decisions [45]. On the other hand, interventions have successfully linked MSM with PrEP care in educational and supportive settings. For instance, a study conducted in an HIV clinic successfully navigated 21% of HIV testing patients to PrEP care, and 16.3% initiated PrEP, by simply providing them with HIV prevention information [10].

The findings in this study are subject to limitations. First of all, as a cross-sectional study, we cannot make causal inferences between the association between PrEP stigma and PrEP cascade. Future longitudinal studies are needed to explore this temporal association. Second, participants’ self-reported risk behaviors may be underreported due to social desirability bias. Their self-reported behaviors may also be subject to recall biases. Although we have accounted for self-reported behaviors as potential confounders in the analyses, unmeasured behavioral factors may introduce biases to the studied associations. Third, due to the limited sample size, we cannot stratify the moderation analyses by other effect modifiers (i.e., race, site locations). However, we have controlled them as confounders when assessing the associations. In addition, the disproportionate sample distribution of the MSM recruited from two cities may lead to biased estimates which may constrain the generalizability. Fourth, as the original purpose was not designed to explore PrEP stigma and PrEP cascade, some unidentified confounders may affect the accuracy of the reported effect sizes. Future studies are needed to specifically explore the studied associations. Fifth, due to the sampling scheme, we only included Black and White MSM in the current analysis. As MSM with other racial/ethnic identities may experience various risk factors and respond differently to the HIV prevention services, findings from the current study may suffer limited generalizability to broader MSM populations in the United States.

PrEP is one of the multiple options for HIV prevention among groups at increased risk for HIV infection. Although multiple clinical trials have endorsed the effectiveness of PrEP in HIV prevention, PrEP is not without cons. For instance, side-effects of antiretroviral therapy, complicated treatment and adherence regime, and the high cost of the drugs all hinder the usage and acceptance of PrEP among health professionals and groups with increased risk of HIV [46,47]. Even in settings with universal public health coverage [48], where the access is not limited by the cost, further efforts should be made to conquer other disadvantages of PrEP use. By the end of 2021, the FDA approved the first injectable long-acting PrEP for adults and adolescents weighing at least 35 kg [49]. These efforts may further empower individuals who have difficulties with daily medical adherence for HIV prevention. Our findings also emphasized the important role of counseling in PrEP care implementation. Proving accurate and non-judgmental counseling assistance would be feasible and effective to engage patients with PrEP care [50].

## 5. Conclusions

Increasing PrEP use is part of the national HIV/AIDS strategy [51], and it is considered one of the four pillars of the Ending the HIV Epidemic initiative in the United States [8]. Despite the Center for Disease Control and Prevention (CDC) and the United States Prevention Service Task Force offering comprehensive guidelines for prescribing and managing PrEP [52,53], and an upward trend being observed across the years since its approval [7], the current PrEP uptake among MSM is still suboptimal to substantially reduce HIV incidence [11,54]. On the other hand, most MSM had participated in HIV testing at different time points [9,55]. Health providers may miss opportunities to provide PrEP to MSM patients who would benefit from using it in HIV prevention services. For instance, health professionals who conduct routine and regular HIV testing can assess patients’ risk behaviors and prescribe PrEP as needed. They can play a critical role in this effort [44,54]. Therefore, a structural-level reform (e.g., educational programs at testing settings, consultation services for MSM at testing settings, education among health professionals) is urgently needed to turn the HIV service encounters into opportunities to facilitate and optimize the PrEP cascade among this group who may benefit the most from PrEP use.

## Figures and Tables

**Table 1:** Demographics and risk behaviors by PrEP cascade status (*n* = 318).

		PrEP Awareness (*n* = 255, 80.19%)		PrEP Willingness (*n* = 260, 81.76%)		PrEP Uptake (*n* = 106, 33.33%)	
	Overall	No	Yes	No	Yes	No	Yes
Age [mean (sd)]	25.86 (4.94)	22.21 (4.05) ****	26.76 (4.72)	25.17 (4.94)	26.01 (4.94)	24.89 (5.00) ****	27.79 (4.21)
Site							
Nashville	236 (74.21%)	25.85% ****	74.15%	19.92%	80.08%	74.15% ****	25.85%
Buffalo	82 (25.79%)	2.44%	97.56%	13.41%	86.59%	45.12%	54.88%
Race							
White	109 (34.28%)	5.50% ****	94.50%	10.09% ***	89.91%	49.54% ****	50.46%
Black	209 (65.72%)	27.27%	72.73%	22.49%	77.51%	75.60%	24.40%
Education							
High School or less	62 (19.50%)	38.71% ****	61.29%	16.13% *	83.87%	88.71% ****	11.29%
Some college	129 (40.57%)	27.13%	72.87%	24.81%	75.19%	74.42%	25.58%
College and above	127 (39.94%)	3.15%	96.85%	12.60%	87.40%	48.03%	51.97%
Having Insurance coverage							
No	58 (18.24%)	34.48% ****	65.52%	20.69%	79.31%	70.69%	29.31%
Yes	260 (81.76%)	16.54%	83.46%	17.69%	82.31%	65.77%	34.23%
Housing							
Stable	241 (75.79%)	12.86% ****	87.14%	16.18%	83.82%	61.83% ***	38.17%
Unstable	77 (24.21%)	41.56%	58.44%	24.68%	75.32%	81.82%	18.18%
Marital status							
Never married	288 (90.57%)	19.79%	80.21%	17.01%	82.99%	68.75% *	31.25%
Ever married	30 (9.43%)	20.00%	80.00%	30.00%	70.00%	46.67%	53.33%
Condomless insertive							
Never	123 (39.68%)	26.02% ***	73.98%	23.58%	76.42%	75.61% **	24.39%
Ever	187 (60.32%)	13.37%	86.63%	14.97%	85.03%	59.36%	40.64%
Condomless receptive							
Never	136 (43.73%)	25.00% ***	75.00%	24.26% *	75.74%	76.47% **	23.53%
Ever	175 (56.27%)	13.14%	86.86%	13.71%	86.29%	57.71%	42.29%
Sex with HIV+ partners							
Never	230 (74.43%)	19.13%	80.87%	20.00%	80.00%	70.87% **	29.13%
Ever	79 (25.57%)	16.46%	83.54%	13.92%	86.08%	50.63%	49.37%
Alcohol use during sex							
Never	132 (41.51%)	33.33% ****	66.67%	22.73%	77.27%	81.06% ****	18.94%
Ever	186 (58.49%)	10.22%	89.78%	15.05%	84.95%	56.45%	43.55%
Recreational drug use during sex							
Never	192 (60.38%)	25.52% ***	74.48%	20.83%	79.17%	71.88% *	28.13%
Ever	126 (39.62%)	11.11%	88.89%	14.29%	85.71%	58.73%	41.27%
Sex position							
Insertive	103 (32.39%)	25.24%	74.76%	23.30%	76.70%	69.90%	30.10%
Receptive	87 (27.36%)	13.79%	86.21%	13.79%	86.21%	59.77%	40.23%
Versatile	128 (40.25%)	19.53%	80.47%	17.19%	82.81%	68.75%	31.25%

Notes: **** < 0.0001, *** < 0.001, ** < 0.01, * < 0.05.

**Table 2:** Mental health, HIV prevention strategy, and HIV-related stigma by PrEP cascade status (*n* = 318).

		PrEP Awareness		PrEP Willingness		PrEP Uptake	
	Overall	No	Yes	No	Yes	No	Yes
Mental Health							
Anxiety (α = 0.93)	7.57 (5.74)	6.75 (7.17)	7.78 (5.32)	6.14 (7.06) *	7.89 (5.36)	7.42 (5.95)	7.87 (5.30)
Depression (α = 0.94)	9.79 (7.47)	7.40 (8.20) **	10.38 (7.17)	7.95 (8.05) *	10.20 (7.28)	9.17 (7.67) *	11.03 (6.92)
Loneliness (α = 0.80)	19.07 (5.08)	17.40 (5.42) **	19.48 (4.91)	16.29 (5.59) ****	19.68(4.75)	18.75 (5.15)	19.71 (4.88)
Perceived Stress (α = 0.89)	18.37 (5.77)	19.53 (4.23) *	18.09 (6.07)	18.72 (5.43)	18.30 (5.85)	18.58 (5.60)	17.95 (6.11)
Suicide (α = 0.83)	5.56 (3.17)	5.86(4.10)	5.49 (2.89)	4.71(2.65) *	5.75(3.24)	5.50 (3.29)	5.68 (2.91)
Internalized homophobia (α = 0.91)	1.78 (1.06)	1.87 (1.16)	1.76(1.04)	1.71 (1.04)	1.80 (1.07)	1.86 (1.12) *	1.63 (0.93)
Resilience (α = 0.96)	2.81 (0.92)	2.50 (1.36) **	2.89(0.76)	2.70 (1.15)	2.83 (0.86)	2.80 (1.03)	2.83 (0.65)
Confidence of condom use (α = 0.88)	4.13 (0.86)	3.88 (1.19) **	4.19 (0.74)	4.01 (1.20)	4.15 (0.76)	4.15 (0.88)	4.08 (0.81)
HIV testing self-efficacy (α = 0.91)	3.90 (0.90)	3.64(1.12) **	3.96 (0.83)	3.86 (1.15)	3.91 (0.84)	3.77 (0.94)	4.14 (0.75) ***
HIV Prevention strategies							
HIV testing in the past 3 m	203 (63.84%)	46.03% **	68.24%	31.03% ****	71.15%	51.42% ****	88.68%
HIV testing in the past 6 m	236 (74.21%)	65.08%	76.47%	53.45% ****	78.85%	66.51% ****	89.62%
HIV testing in the past 12 m	258 (81.13%)	76.19%	82.35%	63.79% ***	85.00%	75.47% ****	92.45%
HIV testing in the past 24 m	274 (86.16%)	76.19% *	88.63%	70.69% ****	89.62%	82.08% **	94.34%
Risk-based HIV test	87 (27.36%)	14.29% ***	30.59%	31.03%	26.54%	24.53%	33.02%
Sero-adaption	241 (75.79%)	38.10% ****	85.10%	68.97%	77.31%	70.75% *	85.85%
Sero-sorting	205 (64.47%)	39.68% ****	70.59%	48.28% **	68.08%	64.15%	65.09%
Stigma							
Internalized PrEP stigma (α = 0.93)	1.99 (4.02)	--	--	2.03 (4.06)	1.99 (4.02)	2.99 (4.85) ****	0.59 (1.57)
Vicarious PrEP stigma (α = 0.93)	4.77 (7.69)	--	--	5.88 (7.82)	4.61 (7.68)	5.56 (8.16) *	3.66 (6.86)
Perceived HIV stigma toward MSM (α = 0.94)	28.99 (9.42)	26.33 (10.39)^**^	29.65 (9.07)	25.28 (10.23) ***	29.99 (9.42)	29.17 (9.85)	28.64 (8.54)

Notes: **** < 0.0001, *** < 0.001, ** < 0.01, * < 0.05.

**Table 3:** Association between PrEP stigma and PrEP cascade (PrEP willingness, and PrEP uptake) among men who have sex with men (*n* = 318).

		Internalized Stigma	Vicarious Stigma	Perceived HIV Stigma toward MSM
Willingness to use PrEP	Willingness (Overall)	0.96 (0.91, 1.01)	1.00 (0.89, 1.12)	1.05 (1.00, 1.11)
	Willingness1	0.96 (0.88, 1.04)	1.01 (0.97, 1.06)	0.98 (0.94, 1.01)
	Willingness2	0.90 (0.83, 0.97)	1.00 (0.96, 1.04)	0.99 (0.96, 1.03)
	Willingness3	0.90 (0.83, 0.97)	1.00 (0.97, 1.05)	0.99 (0.96, 1.03)
	Willingness4	0.89 (0.82, 0.97)	1.00 (0.96, 1.04)	1.00 (0.96, 1.03)
	Willingness5	0.88 (0.81, 0.96)	1.00 (0.96, 1.05)	0.99 (0.95, 1.03)
	Willingness6	0.95 (0.88, 1.02)	1.05 (1.01, 1.09)	0.99 (0.96, 1.03)
	Willingness7	0.92 (0.85, 0.99)	1.01 (0.97, 1.05)	0.98 (0.94, 1.01)
	Willingness8	0.84 (0.76, 0.92)	0.98 (0.94, 1.02)	1.01 (0.98, 1.06)
PrEP uptake	Ever take PrEP	0.76 (0.65, 0.89)	0.97 (0.93, 1.01)	0.96 (0.91, 0.99)
	Currently taking PrEP	1.08 (0.75, 1.55)	1.03 (0.94, 1.12)	0.96, 1.13)

Notes: 1. Willingness1: If a friend or your partner(s) finds out you are taking PrEP and might suggest you are at risk for HIV. 2. Willingness2: if cause mild side effects. 3. Willingness3: if see a clinician every 3–6 months. 4. Willingness4: if get a blood test every 3–6 months. 5. Willingness5: if get a regular HIV test every 3–6 months. 6. Willingness6: if charged a co-pay (fee). 7. Willingness7: if it will not work well if you don’t use it daily. 8. Willingness8: if a friend or your partner(s) finds out you are taking PrEP and might suggest you are at risk for HIV. Willingness adjusted covariates include race, site, education, age, alcohol, and drug use during sex, condomless sex, sex position, HIV testing, condom use confidence, HIV testing self-efficacy, mental health comorbidity, resilience, and homophobia. Uptake adjusted covariates include race, education, age, substance use during sex, insurance, HIV testing, condomless sex, HIV testing, condom use confidence, HIV testing self-efficacy, mental health comorbidity, and resilience.

**Table 4:** Moderation effect of HIV testing at different time points between the association between PrEP stigma and the PrEP cascade (*n* = 318) *.

Moderation Effect	Internalized Stigma (aOR, 95% CI)		Vicarious Stigma (aOR, 95% CI)		Perceived HIV Stigma toward MSM (aOR, 95% CI)	
	PrEP Willingness	PrEP Uptake	PrEP Willingness	PrEP Uptake	PrEP Willingness	PrEP Uptake
24 m testing (*n* = 299)						
0 times	1.03 (0.99, 1.06)	0.99 (0.66, 2.44)	--	--	--	0.95 (0.89, 1.01)
2 times	0.99 (0.97, 1.02)	0.77 (0.65, 0.91)	--	--	--	0.95 (0.91, 0.99)
4 times	0.97 (0.93, 0.99)	0.70 (0.56, 0.87)	--	--	--	0.95 (0.91, 0.99)
6 times	0.94 (0.89, 0.99)	0.63 (0.44, 0.90)	--	--	--	0.95 (0.90, 1.01)
8 times	0.91 (0.84, 0.98)	0.57 (0.34, 0.95)	--	--	--	0.96 (0.89, 1.03)
Trend test ^$^	p < 0.0001	p < 0.0001	p < 0.0001	p < 0.0001	p < 0.0001	p < 0.0001
12 m testing (*n* = 281)						
0 times	1.03 (1.00, 1.06)	0.84 (0.66, 1.07)	--	1.02 (0.94, 1.10)	--	0.98 (0.91, 1.04)
1 times	1.00 (0.98, 1.02)	0.79 (0.66, 0.94)	--	1.00 (0.94, 1.06)	--	0.97 (0.92, 1.02)
3 times	0.94 (0.91,0.99)	0.69 (0.53, 0.89)	--	0.96 (0.92, 1.00)	--	0.96 (0.92, 1.00)
5 times	0.89 (0.83, 0.96)	0.60 (0.37, 0.97)	--	0.92 (0.85, 0.99)	--	0.94 (0.88, 1.01)
Trend test ^$^	p < 0.001	p < 0.0001	p < 0.001	p < 0.0001	p < 0.001	p < 0.0001
6 m testing (*n* = 255)						
0 times	1.02 (0.99, 1.05)	0.86 (0.70, 0.95)	--	--	--	0.98 (0.93, 1.04)
2 times	0.96 (0.93, 0.99)	0.65 (0.51, 0.84)	--	--	--	0.96 (0.92, 1.00)
4 times	0.90 (0.85, 0.96)	0.50 (0.79, 0.85)	--	--	--	0.93 (0.87, 1.00)
5 times	0.87 (0.81, 0.94)	0.43 (0.22, 0.87)	--	--	--	0.92 (0.83, 1.02)
Trend test ^$^	p < 0.0001	p < 0.0001	p < 0.0001	p < 0.0001	p < 0.0001	p < 0.0001
3 m testing (*n* = 219)						
0 times	1.03 (1.00, 1.07)	0.80 (0.62,1.04)	--	1.04 (0.97, 1.11)	--	0.99 (0.94, 1.05)
1 times	0.97 (0.95, 0.99)	0.71 (0.60, 0.85)	--	0.96 (0.91, 1.00)	--	0.96 (0.92, 1.00)
2 times	0.91 (0.88, 0.95)	0.64 (0.48, 0.85)	--	0.89 (0.82, 0.96)	--	0.93 (0.87, 0.99)
3 times	0.87 (0.81, 0.92)	0.58 (0.37, 0.90)	--	0.83 (0.73, 0.95)	--	0.99 (0.82, 0.99)
Trend test ^$^	p < 0.0001	p < 0.0001	p < 0.0001	p < 0.0001	p < 0.0001	p < 0.0001

Notes: * *Willingness* adjusted covariates include: race, site, education, age, alcohol and drug use during sex, condomless sex, sex position, condom use confidence, HIV testing self-efficacy, mental health comorbidity, resilience, homophobia; *Uptake* adjusted covariates include: race, site, education, age, substance use during sex, insurance, HIV testing, condomless sex, HIV testing, condom use confidence, HIV testing self-efficacy, mental health comorbidity, resilience. ***^$^*** Exact p-value is calculated by 10,000 Monte Carlo permutations using the Jonckheere–Terpstra test.

## Data Availability

Limited de-identified raw data available from the corresponding author upon reasonable request.

## Appendix A

**Table A1:** Scales for Key Measurements.

Key Domains	Scale	Sample Questions	Cronbach’s Alpha
Anxiety	7-item Generalized Anxiety Disorder Assessment [20]	“Have you been feeling nervous, anxious, or on edge in the past four weeks?”	α = 0.93
Depression	9-item Patient Health Questionnaire [21,22]	“In the past four weeks, how often did you feel little interest or pleasure in doing things?”	α = 0.94
Loneliness	University of California at Los Angeles Loneliness Scale [23]	“I feel left out.”	α = 0.80
Perceived stress	Perceived Stress Scale [24]	“How often have you been upset because of something that happened unexpectedly?”	α = 0.89
Suicide	A four-question scale adapted from validated studies [18,25]	“Have you ever thought about or attempted to kill yourself?”	α = 0.83
Internalized homophobia	a four-item Internalized Homophobia Scale [26]	“Sometimes I dislike myself for being gay or bisexual.”	α = 0.91
Resilience	10-item Conner-Davidson Resilience Scale [27,28]	“I am able to adapt to change”	α = 0.88
Self-Efficacy for condom use	Condom use Self-Efficacy Scale [29,30]	“I would feel comfortable discussing condom use with a potential partner before we engaged in sex.”	α = 0.88
self-efficacy for HIV testing	HIV testing self-efficacy [31]	“Knowing where you can go for an HIV test”, “Getting tested for HIV at least every 3-6 months.”	α = 0.91
PrEP and HIV related stigma	Internalized PrEP stigma [32]	“I should avoid taking PrEP because it is only for slutty people.”	α = 0.93
	Vicarious PrEP stigma [32]	“I’ve seen/heard people not wanting to hang out with folks who are taking PrEP.”	α = 0.93
	Perceived HIV stigma toward MSM [32]	“People I care about would stop being in touch with me after if I had HIV.”	α = 0.94
